# Electrospun Nanofibers for Tissue Engineering with Drug Loading and Release

**DOI:** 10.3390/pharmaceutics11040182

**Published:** 2019-04-15

**Authors:** Kaiqiang Ye, Haizhu Kuang, Zhengwei You, Yosry Morsi, Xiumei Mo

**Affiliations:** 1 State Key Laboratory for Modification of Chemical Fibers and Polymer Materials, College of Chemistry, Chemical Engineering and Biotechnology, Donghua University, Shanghai 201620, China; yekaiqiang91@gmail.com (K.Y.); kuanghaizhu@gmail.com (H.K.); 2 State Key Laboratory for Modification of Chemical Fibers and Polymer Materials, College of Materials Science and Engineering, Donghua University, Shanghai 201620, China; zyou@dhu.edu.cn; 3 Faculty of Engineering and Industrial Sciences, Swinburne University of Technology, Boroondara, VIC 3122, Australia; ymorsi@swin.edu.au

**Keywords:** electrospinning, tissue engineering, drug delivery

## Abstract

Electrospinning technologies have been applied in the field of tissue engineering as materials, with nanoscale-structures and high porosity, can be easily prepared via this method to bio-mimic the natural extracellular matrix (ECM). Tissue engineering aims to fabricate functional biomaterials for the repairment and regeneration of defective tissue. In addition to the structural simulation for accelerating the repair process and achieving a high-quality regeneration, the combination of biomaterials and bioactive molecules is required for an ideal tissue-engineering scaffold. Due to the diversity in materials and method selection for electrospinning, a great flexibility in drug delivery systems can be achieved. Various drugs including antibiotic agents, vitamins, peptides, and proteins can be incorporated into electrospun scaffolds using different electrospinning techniques and drug-loading methods. This is a review of recent research on electrospun nanofibrous scaffolds for tissue-engineering applications, the development of preparation methods, and the delivery of various bioactive molecules. These studies are based on the fabrication of electrospun biomaterials for the repair of blood vessels, nerve tissues, cartilage, bone defects, and the treatment of aneurysms and skin wounds, as well as their applications related to oral mucosa and dental fields. In these studies, due to the optimal selection of drugs and loading methods based on electrospinning, in vitro and in vivo experiments demonstrated that these scaffolds exhibited desirable effects for the repair and treatment of damaged tissue and, thus, have excellent potential for clinical application.

## 1. Introduction

Electrospinning technology has been widely utilized for the preparation of tissue-engineering scaffolds [1]. The set-up of a typical electrospinning apparatus mainly consists of a spinneret (e.g., a medical injector with a blunt tip), a boost pump for controlling the extrusion rate of a polymer solution, a direct-current electric field, and a grounded collector [2]. The electrospinning technique applies electrostatic principles to fabricate electrospun nanofibers [3]. Generally, in the electrospinning process, a polymer solution generates a cone-shaped drop beneath the needle under a strong electric field; then, the polymer drops overcome the surface tension to eject polymer nanofibers into the low electric field [4,5]. Using electrospinning, various polymers including synthetic and natural materials (as well as blends of the two in consideration of the mechanical properties) can be fabricated as diverse tissue-engineering scaffolds possessing nanofibrous structure. Scaffolds prepared by electrospinning have tremendous advantages for tissue engineering, such as a large specific surface area, high porosity, extracellular matrix (ECM)-biomimetic structures, and better biocompatibility [6,7,8,9,10]. Furthermore, the ECM can bind, release, and activate signaling molecules and can also modulate the cell’s reaction to soluble factors [11]. In order to functionalize the scaffolds for the promotion of cell adhesion, proliferation, and differentiation, nanofibers of the scaffold can be loaded with many bioactive substances, such as proteins, peptides, and small-molecule drugs [12,13,14,15,16,17,18]. Therefore, electrospun scaffolds have remarkable advantages in both ECM-biomimetic structures and the loading of bioactive substances. Electrospun drug delivery scaffolds have emerged as essential applications in the biomedical field [19].

The diversity in material selection and preparation methods gives electrospun scaffolds great flexibility for the application of drug delivery [20]. By utilizing different kinds of electrospinning methods, drugs can be incorporated into electrospun scaffolds in many ways, such as coatings, embedded loading, and encapsulated loading (coaxial and emulsion electrospinning). Traditional electrospinning is the simplest method to prepare electrospun scaffolds. In this method, polymers are directly dissolved into solvent to obtain the electrospinning solution and fabricate the scaffolds. In traditional electrospinning, drugs can be incorporated into the scaffolds either by blending or surface modification. In blending electrospinning, drugs and polymers are simultaneously dissolved in the solvent. Later on, this blended solution is used to fabricate the scaffold [21]. In surface modification, electrospun scaffolds are prepared firstly, then drugs are loaded onto the scaffolds via physical adsorption, covalent binding, and other surface-treatment methods [22]. Accordingly, drugs incorporated into scaffolds via different methods exhibit diverse release characteristics. In blending, the release rate mainly depends on the properties of polymers and drugs in the physiological environment. In physical adsorption, the drug usually shows a faster release rate because of the weaker interaction between drugs and the surface of scaffolds, such as electrostatic adsorption, hydrogen bonding, and hydrophobic interactions [23]. However, drugs covalently bound to the scaffolds hardly release from the scaffolds unless the surface polymers are degraded [24].

Coaxial electrospinning is a special method to generate nanofibers with a core–shell structure. Using a coaxial configuration, two separate polymer solutions (core and shell solutions) flow through two different but coaxial aisles. Core and shell solutions are simultaneously pushed into the configuration. With the slow push of a syringe, the drops are jetted out to form nanofibers under a strong electric field. This technique attracted great attention due to its potential applications in controlled drug delivery [25,26,27]. In coaxial electrospinning, the shell solutions should have spinnability to form the main part of the nanofibers. However, the core solutions have no need for spinnability; thus, some substances without spinnability, such as drugs, proteins, and some bioactive substances, can be incorporated into the core structure of nanofibers by dissolving in the core solution. In this way, these drugs and bioactive substances can be released from the nanofibers in a sustained manner. Therefore, coaxial electrospinning can prepare electrospun scaffolds with bioactivity and functionality. This method is widely used for the preparation of functional nanofibrous scaffolds. A controlled release of functional molecules from the core layer of core–shell scaffolds loaded with drugs, bio-macromolecules, and growth factors could sustainably exert biological functions. For example, tetracycline hydrochloride encapsulated into electrospun core–shell nanofibers showed a sustained release, and exhibited great antibacterial capability, as shown in *Escherichia coli* growth-inhibiting tests [28]. ZnO and Zn acetate nanoparticles were embedded in polycaprolactone coaxial-fiber and uniaxial-fiber matrices to develop potential antibacterial nanocomposite wound dressings [29]. In order to guide tissue regeneration in an infectious environment, coaxial electrospinning was firstly conducted to fabricate dual drug-loaded fiber mats with a core/shell structure. Naringin-loaded polyvinylpyrrolidone was designed as a core fiber to enrich tissue regeneration, and metronidazole-loaded poly(lactic-*co*-glycolic acid) was designed as a shell fiber to inhibit bacterial growth [30].

In addition, nanofibers prepared via the emulsion electrospinning possess the analogous core–shell structure generated by coaxial electrospinning. Therefore, emulsion electrospinning can also be used to prepare core–shell nanofibers for loading and releasing drugs, proteins, and growth factors. However, the process and needle used for emulsion electrospinning are different compared to coaxial electrospinning. Emulsion electrospinning has no need for a coaxial configuration, and the spinning solution is made into a uniform emulsifying solution by blending emulsifier into a polymer–drug mixed solution. However, the polymers and drugs are dissolved in different solvents before blending. Then, the solution is used for the electrospinning process via a single syringe needle as in traditional electrospinning. Finally, nanofibers with multicore or core–shell structures can be obtained. In order to verify the controlled delivery of drugs from this novel type of tissue-engineering scaffold, rhodamine B and bovine serum albumin (BSA) were incorporated into nanofibers via emulsion electrospinning [31]. In vitro dual drug-release studies of different types of electrospun mats indicated that the emulsion electrospun mats had the most desirable controllable release behaviors.

Therefore, electrospun scaffolds, due to their ECM-biomimetic structure and diversity of drug delivery applications, are widely applied in the field of tissue engineering [32]. In the upcoming section, we further introduce their specific applications, such as aneurysm treatment, nerve tissue engineering, vascular tissue engineering, wound dressing, and bone tissue engineering.

## 2. Electrospun Scaffolds for Aneurysm Treatment

**Figure 1:**
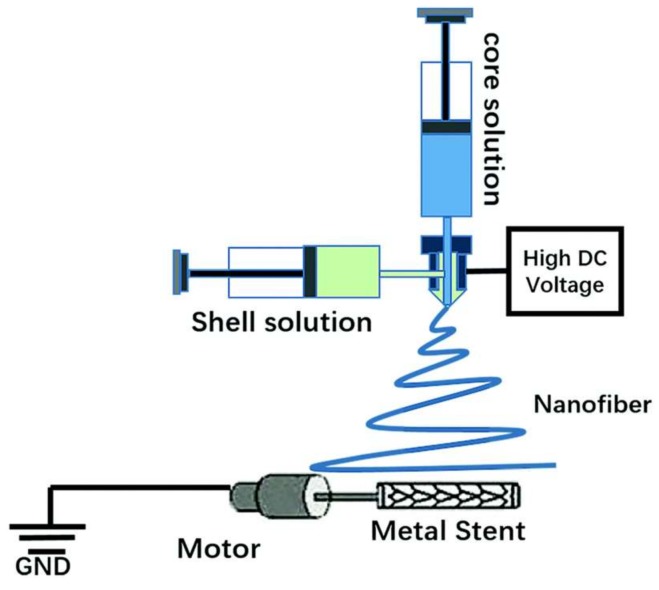
Schematic diagram of the set-up for coaxial electrospinning [39]. Reproduced by permission of The Royal Society of Chemistry (RSC) on behalf of the Centre National de la Recherche Scientifique (CNRS) and the RSC, 2017.

Intracranial aneurysms represent one of the most common cerebrovascular diseases, and they cause serious healthcare problems, such as brain damage, hemorrhagic stroke, and even death [33,34]. Many studies demonstrated that a covered stent is a desirable solution to treat aneurysms [35,36]. However, the common problem is that the stent-graft easily causes platelet aggregation when implanted into the blood vessel, leading to thrombosis and intimal hyperplasia. Rapid endothelialization, antithrombotic coatings, and anti-clotting drugs are favorable methods to avoid thrombosis. A covered stent is formed by a covered sheet wrapping a metallic stent, where the anticoagulation properties of the covered sheet play a crucial role in avoiding thrombosis of the covered stent. The emulsion electrospinning technique was used to fabricate heparin-loaded poly(l-lactide-*co*-caprolactone) (PLCL) core–shell nanofibers as an anticoagulant cover sheet. Heparin aqueous solution and emulsifier Span-80 were added dropwise into dichloromethane while stirring until a uniform water-in-oil emulsion formed. PLCL was then added into the emulsion for electrospinning to fabricate PLCL/heparin core–shell nanofibers. The covered stent effectively separated the aneurysm dome in the bloodstream of the rabbit model [37]. In addition, vascular endothelial growth factor (VEGF) was mixed with heparin and loaded into the core of a PLCL nanofiber via emulsion electrospinning to promote rapid endothelialization [38]. An aqueous solution of VEGF, heparin, and Span-80 was mixed in dichloromethane to form a uniform water-in-oil emulsion via consistent stirring. PLCL was then dissolved in the emulsion to obtain a uniform electrospinning solution. Finally, the solution was electrospun to obtain PLCL–Hep–VEGF scaffolds. The release of heparin and VEGF lasted for more than 30 days. That sustained release of heparin and VEGF enhanced cell proliferation and the spread of pig iliac endothelial cells onto the stent. Rosuvastatin calcium can facilitate endothelialization with the ability to enhance the adhesion and proliferation of vascular endothelial cells (VECs). A novel type of covered stent was prepared via coaxial electrospinning to cover the stent with heparin and rosuvastatin calcium-loaded PLCL nanofibers [39]. The fabrication process of the covered stent is shown in Figure 1, showing heparin and rosuvastatin calcium as the core solution and PLCL as the shell solution. The release curves of heparin and rosuvastatin calcium have two stages, including an initial release that occurred during the first 48 h and a slow release that lasted more than 45 days. The covered stent showed remarkable anticoagulation ability, and the HUVECs proliferated well on the covered stent, due to the sustained release of rosuvastatin calcium and heparin from the PLCL coaxial nanofibers.

## 3. Electrospun Scaffolds for Nerve Tissue Engineering

**Figure 2:**
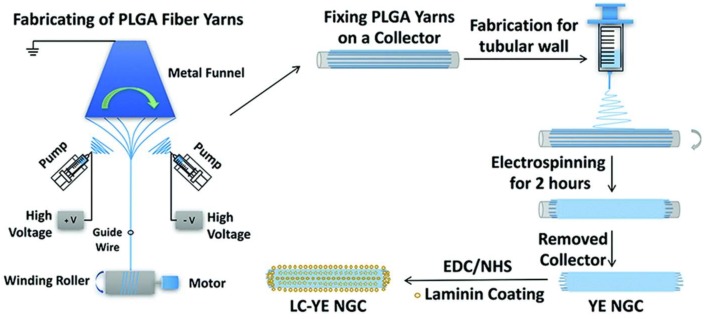
A schematic diagram of the fabrication of the laminin-coated and yarn-encapsulated PLGA (LC-YE-PLGA) nerve guidance conduit (YE NGC: yarn-encapsulated nerve guidance conduit; LC-YE NGC: laminin-coated and yarn-encapsulated nerve guidance conduit) [49]. Reproduced by permission of The Royal Society of Chemistry, 2017.

Peripheral nerve defects represent a serious clinical issue which results in high morbidity among trauma patients [40]. To address the limited regenerative ability of the human nervous system and the shortage of therapeutic options, nerve tissue engineered graft transplantation is a potential treatment for peripheral nerve injury [41,42]. Conduit scaffolds, cells, and growth factors are the three elements for nerve tissue engineering [43]. Core–shell poly(d,l-lactide-*co*-glycolide) (PLGA) nanofibrous nerve guidance conduits loaded with nerve growth factor (NGF) were fabricated via coaxial electrospinning and were used to construct nerve guidance conduits for a 13-mm rat sciatic nerve defect. [44]. The shell solution was PLGA, and the core solution was β-NGF with 400 mg of PEG dissolved in distilled water. A rotating wheel drum with a speed of 4000 rpm was used to received nanofibers to form an aligned construct. The release of NGF from nanofibers lasted 30 days. After 12-weeks implantation, the regeneration of nerve cells in the PLGA/NGF conduit was superior to other groups. Another type of nerve guidance conduit (NGC) was fabricated via coaxial electrospinning. NGF in silk solution as a core layer was encapsulated in PLCL via coaxial electrospinning. NGF presented a sustained release and remained biologically active over 60 days because NGF was stabilized by silk fibroin (SF) in the core [45]. The graft was implanted across a 15-mm defect in the sciatic nerve of rats to evaluate nerve regeneration. Results of electrophysiological assessment, histochemistry, and electron microscopy at 12 weeks suggested that the released NGF from nanofibers could effectively promote the regeneration of the peripheral nerve. In order to further promote cell differentiation, the nanofibers with PLCL as the shell and BSA/NGF as the core were fabricated via coaxial electrospinning [46]. Sustained release of BSA/NGF from nanofibers was verified, and it promoted the differentiation of rat pheochromocytoma cells (PC12). Monosialoganglioside (GM1) can promote neuronal development, cell growth, and differentiation, and it plays an essential role in neuronal excitability of myelinated and nonmyelinated fibers. It was reported that the combination of GM1 with NGF can enhance the effects of protecting nerve cells. NGF and GM1 were loaded into the PLCL/silk fibroin (PLCL/SF) nanofibers via a coaxial electrospinning technique [47]. NGF reached 55.8 ± 1.6% release at 71 days. Schwann cell (SC) proliferation and pheochromocytoma (PC12) differentiation were enhanced by the synergistic effect of GM1 and NGF. The graft loading with GM1 and NGF performed good nerve function recovery in a rabbit sciatic nerve defect model. Vitamins play an important role in tissue growth and differentiation. The water-soluble vitamin B5 was blended with PLCL/silk solutions to form an electrospinning solution. Then, the electrospinning solution was aligned with nanofiber meshes via electrospinning technique [48]. Vitamin B5 was added to a PLCL/silk solution and constantly stirred overnight to form the electrospinning solution. Nanofibers with aligned structure were collected by a rotating drum collector with a speed of 3000 rpm. Vitamin B5 was released up to 80% in 24 h. This nanofibrous material might have potential applications in nerve repair or regeneration. A laminin-coated and yarn-encapsulated PLGA nerve guidance conduit (LC-YE-PLGA NGC) was fabricated to perform the cooperative effects of a topological structure promoting Schwann cell (SC) proliferation and migration [49]. The PLGA fiber yarns were fabricated through a double-nozzle electrospinning system; then, the PLGA fibrous outer layer was collected using a general electrospinning method. Subsequently, laminin was coated on the yarn-encapsulated PLGA NGC through covalent bonding (Figure 2). The cell growth test showed that SC growth and SC migration were much better in the LC-YE-PLGA NGCs than in those without yarn encapsulation or laminin coating. Polycaprolactone (PCL) and chitosan were blended and fabricated to form nanofibrous scaffolds via electrospinning. Taking advantage of the amine groups on the chitosan, the surface of the scaffolds was functionalized with laminin via carbodiimide-based cross-linking. Schwann cells grew well on PCL–chitosan scaffolds with excellent mechanical and surface properties [50]. NGF and GDNF were encapsulated in poly(d,l-lactic acid) (PDLLA) and poly(lactic-*co*-glycolic acid) (PLGA) nanofibers, respectively, via a dual-source dual-power (DS-DP) emulsion electrospinning technique. Scaffolds were developed providing dual GF delivery, and sustained release of both types of GFs was also achieved [51].

## 4. Electrospun Scaffolds for Vascular Tissue Engineering

**Figure 3:**
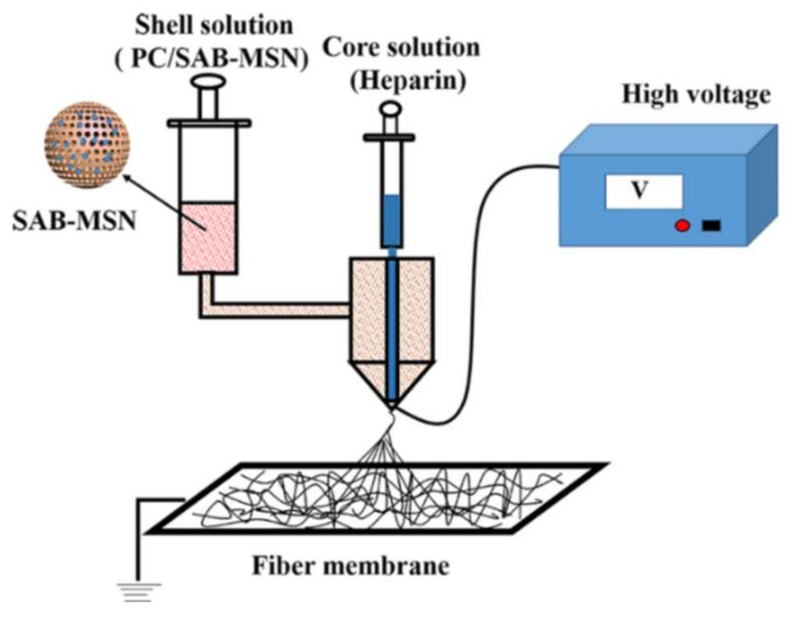
Process of fabricating the core (heparin)−shell (PC/SAB-MSN) fiber. Reprinted with permission from [57] Copyright (2018) American Chemical Society.

Coronary heart and peripheral vascular diseases are now the leading cause of death worldwide [52]. Autologous and allogeneic vascular transplantation has limitations caused by many factors such as donor-site morbidity and the shortage of donors in clinic treatment. Developing artificial blood vessels shows increasing significance [53]. Electrospinning is an ideal technique to prepare grafts for small-diameter blood vessels. Heparin-loaded PLCL nanofibers were prepared via a coaxial electrospinning technique, fabricating tubular grafts with an inner diameter of 4 mm [54]. The release of heparin experienced two stages: an initial burst release of 50% followed by continuous release up to 72% from day 2 to 14. Evaluation with a canine artery model demonstrated that heparin loading could greatly enhance the patency rate of small-diameter grafts. To promote endothelial progenitor cell proliferation, vascular endothelial growth factors (VEGFs) are often loaded into scaffolds. Heparin and VEGF were encapsulated into a PLCL nanofiber via emulsion electrospinning to construct vascular grafts for anticoagulation and rapid endothelization [55]. Heparin/VEGF aqueous solution and Span-80 were added dropwise into methylene dichloride and stirred magnetically to form a uniform water-in-oil emulsion. Then, PLCL was dissolved in the emulsion and stirred overnight to obtain the electrospinning solution. Finally, electrospun vascular grafts were prepared using the electrospinning solution. Heparin and VEGF exhibited sustained release for 29 days. The controlled release of heparin and VEGF from the grafts showed good capability for anticoagulation and promoted EPC growth. Platelet-rich growth factor (PRGF) was added into PLCL/SF solutions at a concentration of 20 mg/mL to obtain the electrospinning solution; then, a tubular graft 4 nm in diameter was prepared via an electrospinning process. The graft promoted fast SMC growth and cell penetration into grafts [56]. Salvianolic acid B (SAB), a traditional Chinese plant medicine, can promote the proliferation and migration of endothelial cells. Heparin was dissolved in reverse osmosis (RO) water as a core solution. PLCL and collagen were dissolved in a solution of 1,1,1,3,3,3-hexafluoro-2-propanol (HFIP) with SAB-MSN as a shell solution. Heparin and SAB-MSN were separately encapsulated into the core and the shell of nanofibers via coaxial electrospinning to construct an inner layer of a small-diameter blood vessel graft. The electrospinning process is shown in Figure 3 [57]. SAB was gently released, and no burst release was observed; this is because SAB was adsorbed onto the MSN and blended into the coaxial fiber shell. The total release of 56% was attained within 30 days. The release of heparin was observed, with an initial burst followed by a steady increase up to 30 days. At the end of the test, the cumulative amount of heparin released was 68%. Assessment of the graft in a rat subcutaneous embedding model demonstrated that it possessed good biocompatibility and did not cause significant immune responses, suggesting that the graft is promising for preventing acute thrombosis and for promoting rapid endothelialization. Some findings provide evidence that surface-immobilized growth factors display enhanced stability and induce prolonged function. PCL nano- or microfibers were produced via electrospinning, and they were coated in a radio frequency (RF) plasma process to induce an oxygen functional hydrocarbon layer. Implemented carboxylic acid groups were converted into amine-reactive esters and covalently coupled to VEGF by forming stable amide bonds. Endothelial cell number was significantly enhanced on VEGF-functionalized scaffolds compared to native PCL scaffolds [58].

## 5. Electrospun Scaffolds for Wound Dressing Application

**Figure 4:**
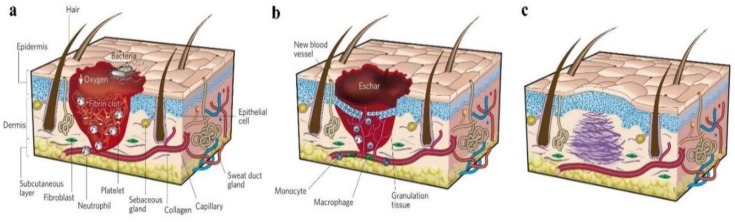
Classic stages of wound repair. There are three classic stages of wound repair: inflammation (**a**), new tissue formation (**b**), and remodeling (**c**) [59]. Reproduced with permission from Springer Nature, 2008.

Skin wounds are a common issue for surgeons. Clinically, the injured skin should be disposed of and carefully protected with wound dressing immediately. Figure 4 exhibits the classic stages of wound repair. There are three classic stages of wound repair: inflammation (a), new tissue formation (b), and remodeling (c) [59]. The effective promotion of the healing of wounds and the prevention of any infection are the functions of wound dressing. Traditional wound dressings are no longer applicable for the required high-quality healing of heavy and chronic skin wounds [60]. In recent years, wound dressings fabricated via electrospinning methods have received a lot of attention in tissue engineering. Electrospinning is a simple and effective technology for preparing materials with a nanofibrous structure which is similar to the ECM. Wound dressing with this structure was proven to promote the repair of injured skin [61,62,63]. This is because the electrospun mats possess nanofibrous porous webs, which are not only suitable for the volatilization of tissue fluid, but also for the permeation of oxygen from the external environment. In addition, many growth factors and antibacterial substances can be loaded onto the electrospun wound dressings in various ways during the fabrication process.

Generally, electrospun wound dressings are fabricated through a typical electrospinning process to obtain membrane materials. Electrospinning solutions were prepared by dissolving single polymers or by blending natural and synthetic polymers; in some cases, the antibacterial substances could be directly mixed into the solutions [64,65]. Moreover, the healing of skin wounds is a complex biological process. In this process, many cellular pathways are activated to regulate cell behaviors to promote the repair of the wound and reduce the probability of infection [66,67]. Furthermore, different growth factors and important active substances in the wound healing process attracted attention in wound dressing applications, such as epidermal growth factor (EGF), platelet-derived growth factor (PDGF), fibroblast growth factor (FGF), transforming growth factor (TGF), insulin-like growth factor (IGF), and human growth hormone and granulocyte-macrophage colony-stimulating factor (GM-CSF) [60]. Other important active compounds in the healing of a wound are vitamins A, C, and E, zinc, iodine, silver nanoparticles, and copper minerals. Therefore, in many studies, there was a growing tendency to incorporate growth factors or antibacterial molecules into the electrospun materials for enhancing the healing quality of wounds [68,69,70,71,72].

Specifically, Sheng et al. [73] prepared a novel vitamin E (VE)-loaded silk fibroin (SF) nanofiber mat for skin care application. Studies suggested that VE possesses antioxidant and skin barrier stabilizing properties, which make VE a suitable agent for skin protection. In this study, water-soluble VE, RRR-α-tocopherol polyethylene glycol 1000 succinate (VE TPGS), was incorporated into SF nanofibers to investigate its potential application in skin care and tissue regeneration. An in vitro study showed that VE TPGS exhibited sustained-release behavior from the nanofiber mats in a physiological environment. In addition, cell experiments demonstrated that nanofibers containing VE TPGS promoted the proliferation of mouse skin fibroblasts (L929 cells) and protected the cell from oxidation.

El-Aassar et al. [74] developed electrospun polyvinyl alcohol (PVA)/pluronic F127 (Plur)/polyethyleneimine (PEI) composite mats containing titanium dioxide nanoparticles (TiO_2_ NPs) for wound dressing application. Titanium ions having wound-healing and antimicrobial properties could be released from TiO_2_ nanoparticles in a slow way, so as to accelerate the wound healing. In this study, TiO_2_ nanoparticles were used as an antimicrobial agent by directly blending them into the electrospinning solution, and antibacterial tests demonstrated that the fabricated PVA-Plur-PEI/TiO_2_ nanofibers exhibited better bactericidal activity than PVA-Plur-PEI nanofibers.

**Figure 5:**
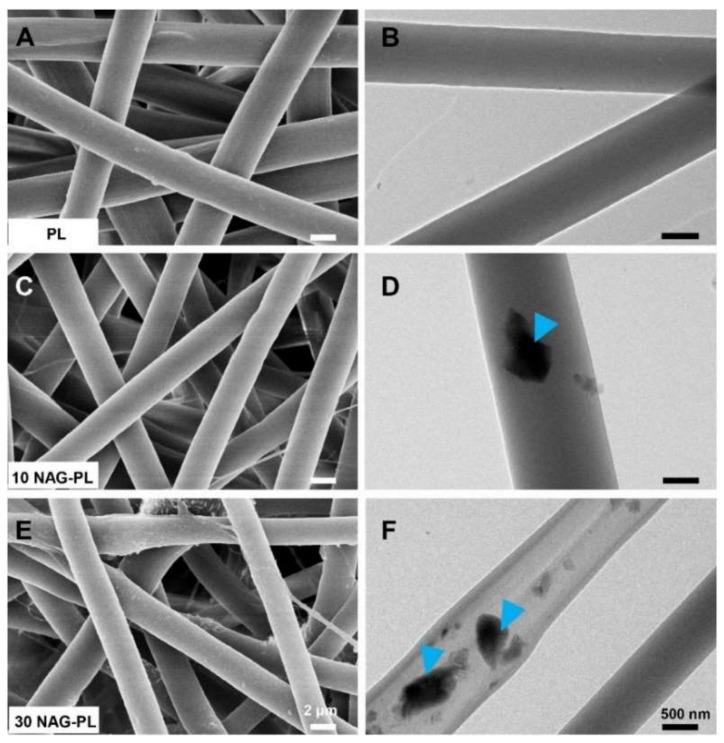
SEM (**A**,**C**,**E**) and TEM (**B**,**D**,**F**) images of the composite electrospun scaffolds with different contents of NAG bioceramic particles: (**A**,**B**) pure polymer (PL); (**C**,**D**) polymer with 10% NAG bioceramic particles (10 NAG-PL); (**E**,**F**) polymer with 30% NAG bioceramic particles (30 NAG-PL). Blue arrows identify the NAG bioceramic particles which were well embedded inside polymer fibers [75]. Reproduced with permission from Elsevier, 2017.

Lv et al. [75] reported an electrospun poly(caprolactone) (PCL)/gelatin scaffold containing silicate-based bioceramic particles (Nagelschmidtite, NAGEL, Ca_7_P_2_Si_2_O_16_) for wound healing. In this study, via a co-electrospinning process, the NAGEL bioceramic particles could be uniformly incorporated into PCL/gelatin fibers, and, with the degradation of the scaffold, the Si ions could be released from the fibers in a sustained way. Figure 5 shows the SEM and TEM images of electrospun fibers containing different amounts of NAGEL bioceramic particles which were well embedded inside polymer fibers [75]. Human umbilical vein endothelial cells (HUVECs) and human keratinocytes (HaCaTs) were cultured into the scaffolds, and cell tests indicated that the scaffolds could significantly promote cell adhesion, proliferation, and migration. Wound healing assessment also displayed that wound sites repaired by these scaffolds exhibited desirable healing results in the aspects of angiogenesis, collagen deposition, re-epithelialization, and inhibiting an inflammation reaction. Furthermore, the mechanism of the high-quality healing of the bioceramic/polymer composite biomaterial was identified as being related to the activation of the epithelial-to-mesenchymal transition (EMT) and endothelial-to-mesenchymal transition (EndMT) pathway.

As a result of incorporating various bioactive molecules into the nanofibers, these novel electrospun biomaterials provided strategies to design functional dressings for the rapid and high-quality healing of skin wounds.

## 6. Electrospun Scaffolds for Tendon Tissue Engineering

Surgical repair utilizing autografts, allografts, xenografts, tendon prostheses, and suture techniques are the main therapies in the current approach to the treatment of tendon injuries [76]. However, tendon grafts clinically used in surgical treatments fail to meet the demands of adaptability, flexibility, and perpetual remodeling. To address these problems, tissue-engineering scaffolds based on electrospun fibers provide potential alternatives for the treatment and regeneration of damaged tendon tissue. The combination of synthetic polymers and natural materials was an easy method to obtain electrospun scaffolds with both biocompatibility and excellent mechanical strength. Electrospun fibers materials have been investigated in tissue engineering for the potential application of tendon treatment [77,78,79]; these studies revealed that electrospun composite scaffolds for tendon tissue engineering performed much better than conventional tendon materials in the aspects of biocompatibility, cell adhesion, proliferation, and mechanical properties.

A desirable scaffold that possesses both suitable mechanical properties and biological signals is required for tendon tissue engineering [80]. In recent years, novel spinning approaches were used to fabricate scaffolds mimicking the hierarchical structures of natural tendon tissue [81,82], and to accelerate the healing of tendon defects and further induce tendon regeneration. In addition to varying the preparation method, tendon scaffolds containing various bioactive molecules were also fabricated [83,84].

**Figure 6:**
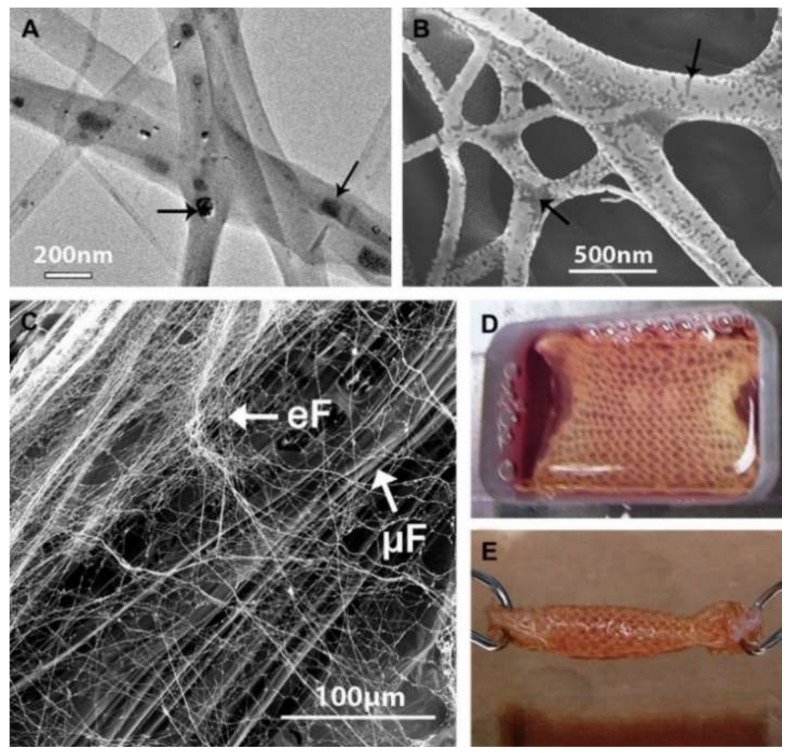
(**A**) TEM and (**B**) back-scattered SEM images of electrospun bFGF-containing PLGA fibers showing the random distribution of proteins (indicated by black arrows) in the fibers; (**C**) SEM image of biohybrid scaffold developed by coating FGF(+) electrospun fibers (eF) on microfibrous knitted silk scaffolds (mF); (**D**,**E**) BMSC-seeded biohybrid scaffold cultured in a custom-made chamber before being rolled up into cylindrical ligament/tendon analogs after seven days of culture [85]. Reproduced with permission from Elsevier, 2010.

Sahoo et al. [85]. developed a hybrid scaffold containing both microfibers and nanofibers. Specifically speaking, the knitted silk scaffolds were firstly prefabricated and coated with an aqueous silk solution; then, the knitted scaffolds were installed onto a rotating collector during electrospinning, and the solution for electrospinning was prepared by dissolving PLGA and basic fibroblast growth factor (bFGF) into HFIP. As a result, the knitted scaffolds were wrapped around the electrospun PLGA ultrafine fibers. In this biohybrid scaffold system, silk microfibers were responsible for enhancing mechanical properties, while PLGA nanofibers, coated on the silk scaffolds, could release bFGF in a sustained manner, which was incorporated into PLGA nanofibers via blending electrospinning. As shown in Figure 6A,B, the bFGF was randomly distributed in the PLGA fibers; Figure 6C shows the biohybrid scaffold developed by coating protein-containing (FGF+ group) electrospun fibers (eF) onto microfibrous knitted silk scaffolds (μF). In cell evaluation, mesenchymal progenitor cell (MPC) was seeded into the biohybrid scaffold. Results showed that the biohybrid scaffold system not only promoted MPC attachment and cell proliferation, but also stimulated the tenogeneic differentiation of seeded MPCs. Figure 6D,E exhibits the BMSC-seeded ligament/tendon analogs after seven days of culture. Moreover, following a three-week co-culture of MPCs and scaffold, the generated tendon analog indicated that the scaffold has potential for repairing tendon defects.

**Figure 7:**
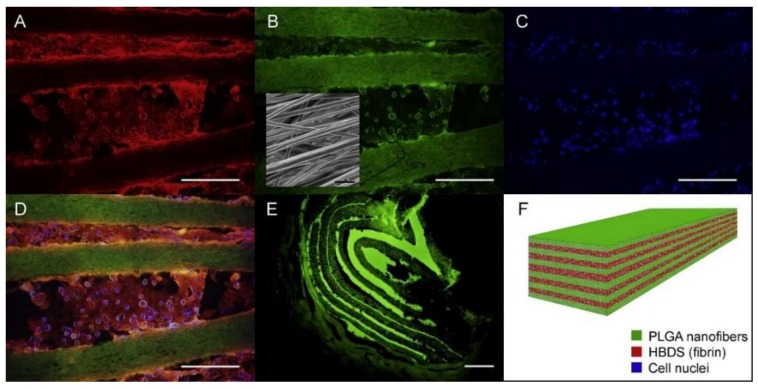
A representative HBDS/nanofiber scaffold with 11 alternating layers of aligned electrospun PLGA nanofiber mats separated by HBDS containing 1 × 10^6^ ASCs is shown. (**A**–**D**) Micrograph showing the HBDS/nanofiber scaffold in vitro; the PLGA is labeled with FITC (green), the HBDS is labeled with Alexa Fluor 546 (red), and the ASC nuclei are labeled with Hoescht 33258 (blue) (scale bar = 200 μm). (**B**, inset) SEM image of the scaffold showing PLGA nanofiber alignment. (**E**) Micrograph showing the HBDS/nanofiber scaffold in vivo nine days after implantation in tendon repair. Eleven alternating layers of PLGA and HBDS can be seen (i.e., six layers of PLGA and five layers of fibrin); the PLGA is labeled with FITC (green) (scale bar = 100 μm). (**F**) A schematic of the layered scaffold is shown [86]. Reproduced with permission from Elsevier, 2013.

Manning et al. [86] reported a scaffold with the capacity for controlled delivery of growth factors and cells for tendon tissue engineering. Platelet-derived growth factor BB (PDGF-BB), together with adipose-derived mesenchymal stem cells (ASCs), was firstly incorporated into a heparin/fibrin-based delivery system (HBDS). Then, the hydrogel was layered with electrospun nanofibers to obtain the resulting composite scaffold. In vitro and in vivo studies verified that this fabricating strategy allowed the novel layered scaffold to simultaneously and effectively deliver growth factors and fulfill cell migration in a controlled manner in the tendon repair environment so as to promote the tendon healing. Figure 7 shows the fluorescence dyed cells on the scaffold and the schematic of the scaffold structure and ingredients.

## 7. Electrospun Scaffolds for Bone Tissue Engineering

**Figure 8:**
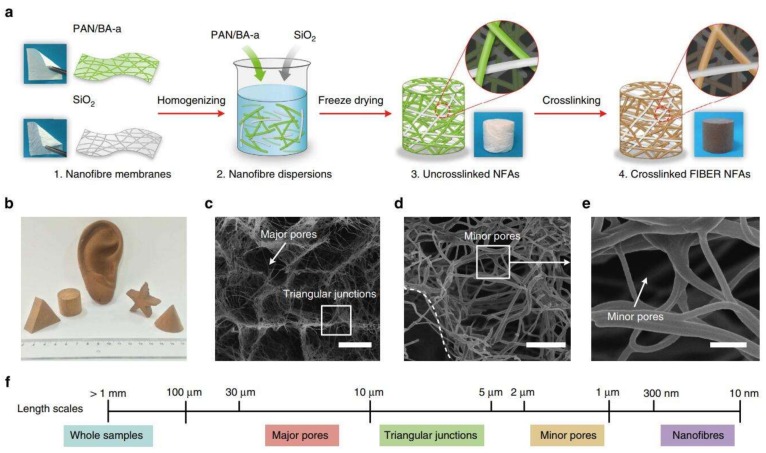
(**a**) Schematic of the synthetic steps. (**b**) The pictures of fabricated scaffolds. (**c**–**e**) SEM images of the electrospun nanofibrous scaffolds. (**f**) Length scales: 20 μm (**c**), 5 μm (**d**), 1 μm (**e**) [92]. Reproduced with permission from Springer Nature, 2014.

Bone tissue-engineering scaffolds are applied to repair bone defects mainly caused by tumors, trauma, osteoporosis, and infection. Due to a limited donor site in autografting treatment, bone tissue engineering aims to produce functional bone scaffolds as alternatives for clinical treatments [87]. An ideal bone scaffold should mimic the architecture of the native ECM to provide a three-dimensional (3D) environment for cell adhesion, proliferation, and differentiation [88]. However, nanofiber materials prepared via traditional electrospinning only possess nanofibrous structure on a two-dimensional level, lacking a 3D porous structure essential for nutrient transport and tissue regeneration; it is estimated that the interconnected spaces >100 μm are required for vascularized bone tissue growth [89]. Recently, emerging strategies were applied to prepare 3D nanofibrous scaffolds based on electrospun nanofibers, which have attracted attention in tissue engineering, especially bone tissue engineering [90,91,92]. Figure 8 shows the typical strategy of the preparation of electrospun 3D nanofibrous scaffolds; this method mainly consists of nanofiber preparation, homogenization, freeze-drying, and cross-linking processes. These scaffolds possess nanofibrous morphologies and interconnected pores. In addition, a prevailing strategy in bone engineering is the combination of growth factors and scaffolds to facilitate the osteogenic differentiation of stem cells in vitro, and bone regeneration in vivo.

Several studies verified the crucial role of growth factors in regulating cell behaviors, such as recruitment, proliferation, and differentiation [93,94]. Currently, bone morphogenetic proteins (BMPs) are the most effective osteoinductive growth factors investigated in bone tissue engineering [24]. In particular, BMP-2 was approved for clinical application by the FDA. Su et al. [95] developed a BMP-2-and dexamethasone (DEX)-loaded core–shell electrospun mat for use in bone tissue engineering. BMP-2 and DEX were successfully incorporated into nanofibers via blending or coaxial electrospinning, and evaluation of release in vitro indicated that coaxial electrospinning nanofibers exhibited better controlled release of the two substances than blending electrospinning nanofibers. Moreover, the BMP-2 and DEX released from nanofibers induced human mesenchymal stromal cells (hMSC) to differentiate into osteogenic cells.

**Figure 9:**
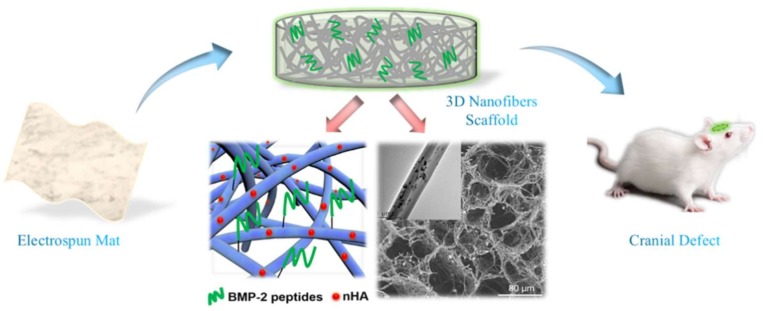
Three-dimensional (3D) electrospun nanofibrous scaffold for rat cranial bone regeneration [23]. Reproduced with permission from Elsevier, 2019.

However, BMP-2 has the disadvantages of rapid enzymatic hydrolysis, ectopic bone formation, immune reactions, and high cost [96,97]. Recently, BMP-2-derived peptides gained much attention as alternative bioactive molecules. The sequences of the peptides were synthesized based on the “wrist” epitope and “knuckle” epitope, which are supposed to bind to BMP receptors [98]. Studies based on BMP-2-derived peptides indicated that these peptides had positive impacts on osteogenic differentiation of stem-cell and bone formation in defects [99,100]. For example, Ye et al. [23] developed a scaffold with a 3D nanofibrous porous structure for bone tissue engineering. In this study, by combining homogenization, freeze-drying, and thermal treatment, nano-hydroxyapatite/PLLA/gelatin (nHA/PLA/GEL) 3D nanofibrous scaffolds were fabricated using pre-prepared electrospun nanofibers. Then, utilizing a polydopamine (pDA)-assisted coating strategy, BMP-2-derived peptides (PEP, sequence: S_[PO4]_KIPKASSVPTELSAISTLYLDDD) were immobilized onto the 3D scaffolds to obtain the resulting nHA/PLA/GEL-PEP 3D nanofibrous scaffolds capable of sustained release of BMP-2 peptides. Figure 9 illustrates the highlights of the study from fabrication to animal experiment. In vitro studies demonstrated that nHA/PLA/GEL-PEP scaffolds promoted the activity of alkaline phosphatase of bone mesenchymal stem cells (BMSCs) and gene expression related to osteogenic differentiation. Moreover, in vivo evaluation was performed using a rat cranial bone defect model, and the results of radiology and histology analysis indicated that this scaffold facilitated bone formation in the defects. Therefore, the scaffold has excellent potential in bone defect repair [23].

## 8. Conclusions

In this review, various scaffolds based on electrospinning for tissue engineering have been discussed, and various strategies for designing novel scaffolds explained. Preparation and delivery methods highlighted indicate that electrospinning technology is a useful tool to fabricate scaffolds with nanofibrous structure. Moreover, bioactive molecules can also be incorporated into the scaffolds via method selection or surface modification for controlling the drug delivery. The ECM-mimicking structure, along with the delivery system based on electrospinning, makes electrospun scaffolds ideal biomaterials for tissue-engineering applications. Key aims going forward should be to address the relationship between mass production and material stability, and the deeper impacts on cells generated by these drug-loaded scaffolds should be illustrated.
